# Finerenone Versus Placebo on Renal Outcomes in Patients with Chronic Kidney Disease and Type 2 Diabetes: A Systematic Review and Meta-Analysis

**DOI:** 10.3390/jcm14186355

**Published:** 2025-09-09

**Authors:** Gerson E. Diaz, Gianfranco H. Mostacero, Brenda Huamaní, Raysa Gutierrez-Rodriguez, Oriana Rivera-Lozada, Judith Yangali-Vicente, Joshuan J. Barboza

**Affiliations:** 1 Escuela de Medicina Humana, Universidad San Ignacio de Loyola, Lima 15024, Peru; gerson.diaz@usil.pe (G.E.D.); gianfrancohmm@gmail.com (G.H.M.); brendahuamanirodriguez@gmail.com (B.H.); 2 Facultad de Salud Pública y Administración, Universidad Peruana Cayetano Heredia, Lima 15102, Peru; raysagutrod@gmail.com; 3 Vicerrectorado de Investigación, Universidad Señor de Sipan, Chiclayo 14002, Peru; riveraoriana@uss.edu.pe; 4 Dirección de Investigación, Universidad Inca Garcilaso de la Vega, Lima 15084, Peru; judith.yangali@uigv.edu.pe; 5 Vicerrectorado de Investigación, Universidad San Ignacio de Loyola, Lima 15024, Peru

**Keywords:** cardiovascular outcomes, chronic kidney disease, finerenone, meta-analysis, type 2 diabetes

## Abstract

**Background/Objectives:** To evaluate the efficacy and safety of finerenone compared to placebo in improving renal outcomes in patients with chronic kidney disease (CKD) and type 2 diabetes (T2D). **Methods:** A systematic review and meta-analysis were conducted, compiling RCTs evaluated the effect of finerenone compared to placebo in patients with CKD and T2D. Inclusion criteria included adults with CKD and T2D. Outcomes included kidney failure, end-stage renal disease (ESKD), and persistently decreased glomerular filtration rate (eGFR). Secondary outcomes included cardiovascular events, hospitalization due to hyperkalemia, and serious adverse events. Pooled relative risks (RRs) and mean differences (MDs) were calculated using a random-effects model. **Results:** Three RCTs with a total of 19,027 patients were included. Finerenone demonstrated a potential reduction in kidney failure risk (RR 0.86, 95% CI: 0.35–2.13) and ESKD (RR 0.82, 95% CI: 0.54–1.23); however, confidence intervals included the null effect. There were no statistically significant differences, as seen in the decrease in eGFR (RR 1.03; 95% CI: 0.27–3.85), but also in mortality due to renal causes (RR 0.62; 95% CI: 0.00–7168.81). Finerenone increased hyperkalemia-related hospitalizations (RR 4.57, 95% CI: 1.07–19.48) but had no significant effect on serious adverse events (RR 0.94, 95% CI: 0.92–0.97) or systolic BP (MD 0.08 mmHg, 95% CI: −0.36 to 0.52). **Conclusions:** Finerenone may provide renal protection in CKD and T2D, though benefits remain uncertain due to wide confidence intervals and study heterogeneity. The increased risk of hyperkalemia warrants careful patient selection and monitoring. Further research is needed to refine its clinical applicability. Review registration: PROSPERO CRD420250642593.

## 1. Introduction

Chronic kidney disease (CKD) is a global health concern with a prevalence rate of approximately 10–15% among adults, with a great likelihood of exacerbating cardiovascular morbidity and mortality, particularly for individuals with type 2 diabetes (T2D) [1]. The co-occurrence of CKD with T2D leads to progressive kidney injury and cardiovascular disease with the potential for heart failure (HF), myocardial infarction, and stroke [2]. The cardiovascular-renal pathophysiological interaction, commonly referred to as cardiorenal syndrome, reflects the need for intervention through targeted treatments to reduce harmful outcomes [3]. Overactivity of the mineralocorticoid receptor (MR) has a central role in the pathophysiology of CKD and cardiovascular disease (CVD) through inflammation and fibrogenic pathways within cardiac and kidney tissues [4]. Conventional steroid MR antagonists, including spironolactone and eplerenone, exhibit effectiveness for managing heart failure but are usually accompanied by a heightened risk of hyperkalemia and kidney injury, precluding their administration to individuals with CKD [5]. A selective, nonsteroidal MR antagonist, finerenone, has been shown to represent a potential beneficial addition to the existing multifaceted strategy for CKD with T2D due to its favorable safety profile and effectiveness for alleviating cardiorenal complications in individuals with CKD and T2D [6].

The effectiveness of finerenone has been thoroughly studied using large-scale clinical trials. The FIDELIO-DKD and FIGARO-DKD trials proved that finerenone decreases the cardiovascular events and kidney failure risk among individuals with CKD and T2D by a significant margin [7]. A pooled analysis of these trials (FIDELITY) reinforced that finerenone decreases cardiovascular events by 14% and kidney events by 23% against placebo [8].

In the FIGARO-DKD trial, finerenone lowered heart failure hospitalization by 32% and improved cardiovascular outcomes with no major increase in the risk of severe hyperkalemia [9]. In the FIDELIO-DKD trial, finerenone showed significant benefits in slowing CKD progression, especially among individuals with severe albuminuria [10].

In light of these encouraging findings, there are nevertheless knowledge gaps concerning the direct effect of finerenone on single cardiovascular and kidney outcomes, especially among high-risk subgroups. The current study will present a holistic meta-analysis on the effectiveness and safety of finerenone across CKD and T2D patients with respect to clinically significant outcomes like kidney failure and sustained eGFR decline. In bringing together evidence from recent trials, the present study hopes to shed more light on finerenone as a cornerstone therapy for individuals situated at the confluence of cardiovascular as well as kidney disease.

## 2. Materials and Methods

This study was a systematic review with meta-analysis. The reference elements for systematic reviews and meta-analyses (PRISMA-2020) informed this review [11]. The PROSPERO registered was indexed in: CRD420250642593.

### 2.1. Search Strategy

A limited initial search was conducted in MEDLINE (via PubMed, U.S. National Library of Medicine, Bethesda, MD, USA) and Embase (Elsevier, Amsterdam, The Netherlands) to identify notable articles on the topic. The text words contained in the titles and abstracts of these articles, along with the index terms used to describe them, were utilized to develop a complete search strategy for MEDLINE (via PubMed, U.S. National Library of Medicine, Bethesda, MD, USA), Scopus (Elsevier, Amsterdam, The Netherlands), Web of Science (Clarivate Analytics, Philadelphia, PA, USA), and Embase (Elsevier, Amsterdam, The Netherlands) (see Supplementary Table S1 for full search details). The search strategy, including all identified keywords and index terms, was adapted for each database and information source. The reference lists of all studies selected for critical appraisal were also screened for additional relevant studies. There was no limitation by language or year of publication. The search covered studies published from database inception to the present to ensure a comprehensive evaluation of the available evidence, particularly considering the approval and widespread clinical testing of Finerenone in recent years. Additional sources of unpublished studies and gray literature included trial registries (e.g., ClinicalTrials.gov).

### 2.2. Eligibility Criteria

Studies that evaluated adult patients (≥18 years) with a confirmed diagnosis of chronic kidney disease (CKD) and type 2 diabetes mellitus (T2D) were included. All patients had controlled blood pressure using standard treatment (e.g., angiotensin-converting enzyme inhibitors [ACEIs] or angiotensin receptor blockers [ARBs]). Patients with renal failure, end-stage kidney disease (ESKD) who presented with severe hyperkalemia at baseline or had serious medical conditions that impaired tolerance to Finerenone treatment were excluded.

Only studies evaluating Finerenone, a selective mineralocorticoid receptor antagonist, administered orally, were included. The intervention consisted of administering doses of 10 or 20 mg daily, titrated based on serum potassium levels and patient tolerance, with a follow-up period of one to three years. Studies that did not clearly describe the dosing protocol or those that used pharmacological combinations, including other mineralocorticoid receptor antagonists, were excluded.

According to current clinical guidelines, the comparator group consisted of patients receiving standard treatment for CKD and T2D. Studies comparing Finerenone with placebo or with standard therapy, excluding mineralocorticoid receptor antagonists, were included, while studies using active comparators (i.e., other therapies targeting mineralocorticoid receptors) were excluded to ensure a direct evaluation of Finerenone’s safety and effectiveness.

Only randomized controlled trials (RCTs) were included, as they provide the highest level of evidence for assessing the effectiveness and safety of interventions. Non-randomized controlled trials, quasi-experimental studies, cohort studies, case-control studies, cross-sectional studies, case series, and individual case reports were excluded. This decision ensured that the analysis focused on high-quality, randomized evidence directly addressing the review question.

### 2.3. Outcomes

The primary renal outcomes assessed included kidney failure, defined as a severe loss of kidney function requiring dialysis or kidney transplantation. Additionally, end-stage kidney disease (ESKD) was evaluated as a distinct outcome, representing the most advanced stage of CKD requiring long-term renal replacement therapy. A sustained decrease in eGFR was analyzed as an indicator of progressive renal function decline, with particular emphasis on patients experiencing a sustained reduction of 40 in eGFR, a recognized threshold for significant worsening of kidney function. Furthermore, renal death was a key variable for measuring the impact of CKD progression and patient survival. Secondary outcomes included cardiovascular and drug safety variables, due to the potential effects of target receptor inhibition on electrolyte balance. The incidence rate of hospitalization for hyperkalemia was considered a serious and fatal complication in patients with CKD. Likewise, the occurrence of any serious adverse event (SAE) was evaluated, including all significant events contributing to death, prolonged hospitalization, and disability. Finally, changes in systolic blood pressure were analyzed in patients stratified by eGFR stage to determine the drug’s blood pressure-lowering effects, considering potential correlations with cardiovascular protection.

### 2.4. Study Selection

After the search, the collected citations were compiled and uploaded to Rayyan (Rayyan Systems Inc., Doha, Qatar), where duplicates were removed. Four reviewers independently checked the titles and abstracts, following established inclusion criteria to identify relevant articles. A pre-screening test was conducted in Rayyan to ensure consistency and quality. Relevant studies were fully retrieved, and their citation details were imported into Endnote 21 (Clarivate Analytics, Philadelphia, PA, USA). The systematic review documented and reported the reasons for excluding full-text studies that did not meet the inclusion criteria. The search results, study selection, and inclusion process were fully reported and presented in a PRISMA flow diagram, following the PRISMA 2020 guidelines.

### 2.5. Data Extraction

After performing electronic searches, all results were compiled into a single library, and duplicates were removed. Three reviewers independently selected studies in two phases. The first screening phase involved evaluating titles and abstracts using the Rayyan platform and applying inclusion and exclusion criteria. Studies that passed this phase were retrieved and analyzed in full text, followed by a second screening based on the inclusion and exclusion criteria, with justifications provided. Eligible studies were included in the systematic review, and data extraction began. In cases of disagreement, a referee reviewer (JJB) was consulted. Data were extracted individually and blinded using a pre-prepared Excel spreadsheet, including author, year of publication, country, study type, number of participants per intervention arm, selection criteria, intervention and control description, and primary and secondary outcomes.

### 2.6. Risk of Bias Assessment

Pairs of reviewers independently and in duplicate evaluated each RCT for potential bias using the Risk of Bias Tool 2 (RoB 2). Disagreements were resolved through discussion with a third reviewer (JJB). The risk of bias for each domain and study was categorized as low, with some concerns, or high.

### 2.7. Data Synthesis

A random-effects model with the inverse variance method was used for all meta-analyses assessing the effects of Finerenone on primary and secondary outcomes compared with placebo. The Paule-Mandel method was applied to calculate between-study tau^2^ variance. Probiotic effects on dichotomous outcomes were reported as relative risks (RRs) with 95% confidence intervals (CIs). A continuity correction method was used to adjust for null events in one or both RCT arms. For meta-analyses involving more than five studies, the Hartung-Knapp method was used. Statistical heterogeneity was assessed using the I^2^ statistic, where values represented low (<30%), moderate (30–60%), and high (>60%) heterogeneity. The metabin function in R (version 3.5.1, meta library) was used for the analysis.

### 2.8. GRADE Assessment

The certainty of evidence for all reported outcomes was assessed by consensus among the authors (JJB, NCG, CQV, HTCh) using the Grading of Recommendations, Assessment, Development, and Evaluations (GRADE) approach. The GRADE methodology evaluated the certainty of evidence across all outcomes, categorizing it as high, moderate, low, or very low. Domains assessed included risk of bias, inconsistency, indirectness, imprecision, and publication bias. The certainty of the evidence was summarized in SoF tables created using GRADEpro GDT (McMaster University, Hamilton, ON, Canada; accessed January 2025).

## 3. Results

### 3.1. Selection of Studies

**Figure 1:**
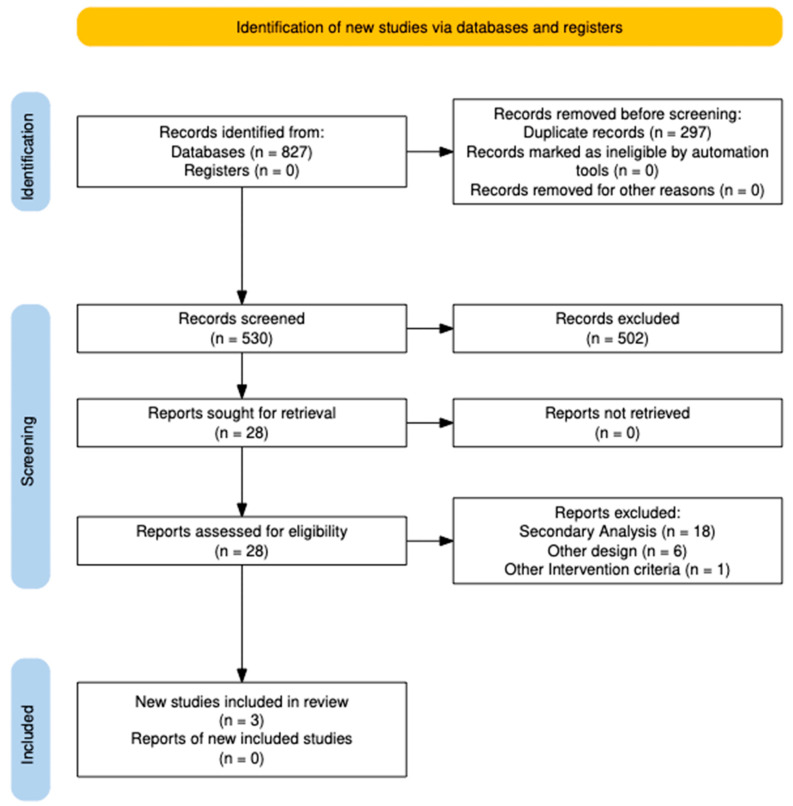
Flow chart selection process.

We evaluated 827 studies in the different databases, of which 297 were duplicates and were removed. Of the remaining 530, 502 were excluded by title and abstract based on eligibility criteria. The remaining 28 studies were sought for full-text retrieval, all successfully obtained. After a detailed eligibility assessment, 25 studies were excluded due to secondary analysis (*n* = 18), different study design (*n* = 6), or failure to meet the intervention criteria (*n* = 1). Finally, three studies [12,13,14] were included in the systematic review (Figure 1).

### 3.2. Characteristics of Included Studies

The included studies demonstrated a high methodological rigor, utilizing randomized controlled trial (RCT) designs across multiple international settings (Table 1). The trials were conducted in various regions, including North America, Europe, Asia, and Latin America, with some spanning over 40 countries. The study population comprised thousands of participants, predominantly adults aged 40 years and older, with chronic conditions such as chronic kidney disease (CKD), type 2 diabetes, and heart failure. The trials ensured a diverse representation of patients, with variations in race, gender distribution, and baseline comorbidities.

Patient characteristics were well-defined, with strict inclusion criteria ensuring homogeneity within study populations (Table 1). Most participants had significant metabolic and cardiovascular risk factors, including long-standing diabetes, hypertension, and renal dysfunction. The estimated glomerular filtration rate (eGFR) was commonly used to stratify patients, with classifications based on moderate to severe albuminuria. Follow-up durations varied but generally extended beyond two years, allowing for assessing long-term efficacy and safety outcomes. Throughout the studies, adherence to the trial regimen remained high, reinforcing the reliability of the reported findings.

Regarding interventions, all studies evaluated finerenone, a selective nonsteroidal mineralocorticoid receptor antagonist, administered orally at doses ranging from 10 mg to 40 mg daily (Table 2). The drug was compared to a placebo, and treatment adherence was maintained through a double-blind design. All participants received standard-of-care therapy, which included renin-angiotensin system (RAS) inhibitors such as angiotensin-converting enzyme (ACE) inhibitors and angiotensin receptor blockers (ARBs). Additionally, many patients were treated with sodium-glucose cotransporter 2 (SGLT2) inhibitors, statins, insulin, and other glucose-lowering therapies.

### 3.3. Risk of Bias Results

The risk of bias analysis for the studies included in this systematic review and meta-analysis on finerenone versus placebo revealed an overall low probability of bias, with some exceptions. Five key domains were assessed using the Risk of Bias 2 (RoB 2) tool (Table 3).

Regarding the domain related to bias in the randomization process (D1), some concerns were identified in the study by [13], suggesting that the allocation method may not have been entirely transparent or that there were baseline imbalances between groups. However, the studies by [12,14] were classified as having a low risk of bias in this aspect, indicating that participant assignment was conducted appropriately, minimizing the potential for bias.

The domain assessing bias due to deviations from the intended intervention (D2) showed a low risk across all studies. This suggests that participants received the intervention as planned, and no significant deviations were detected that could compromise the study outcomes.

Regarding bias for outcome measurement (D4), all exhibited a low risk, indicating that the evaluation was carried out honestly and without systematic disparities in outcome assessment between the intervention and control groups.

Last but not least, bias related to the selection of reported results (D5) was likewise deemed low risk. This shows that there was no indication of data selection or omissions, and that the analyses performed and the results displayed followed the original protocols.

### 3.4. Effect of Finerenone on Primary and Secondary Outcomes

**Figure 2:**
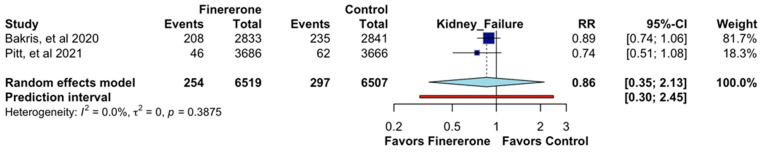
Effect of Finererone on kidney failure. Data from Bakris et al., 2020 [14], and Pitt et al., 2021 [12].

The meta-analysis assessing the effect of finerenone on kidney failure included two randomized controlled trials (RCTs) [12,14], with a total of 13,026 participants. The pooled relative risk (RR) for kidney failure was 0.86 (95% CI: 0.35–2.13; CoE Low; Figure 2), indicating a potential reduction in the risk of kidney failure with finerenone compared to the control group. However, the confidence interval is wide and crosses the null value (RR = 1), suggesting that the effect is not statistically significant. The individual study results show an RR of 0.89 (95% CI: 0.74–1.06) for [14], which contributed 81.7% of the total weight in the analysis, and an RR of 0.74 (95% CI: 0.51–1.08) for [12], which contributed 18.3% of the weight. The heterogeneity analysis indicated no significant between-study variability (I^2^ = 0.0%, τ^2^ = 0, *p* = 0.3875), reinforcing the consistency of findings across studies. Despite the trend favoring finerenone, the broad confidence intervals suggest uncertainty in the actual effect size.

**Figure 3:**
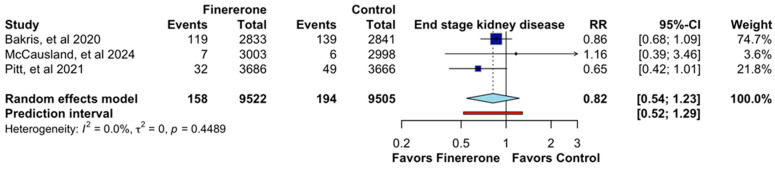
Effect of Finererone on end-stage kidney disease. Data from Bakris et al., 2020 [14], McCausland et al., 2024 [13], and Pitt et al., 2021 [12].

Similarly, the analysis of end-stage kidney disease (ESKD) included three randomized controlled trials (RCTs) [12,13,14], encompassing a total of 19,027 patients. The pooled relative risk (RR) for ESKD was 0.82 (95% CI: 0.54–1.23; CoE Low; Figure 3), suggesting a potential benefit of finerenone in reducing the risk of disease progression compared to the control group. However, as with kidney failure, the confidence interval crosses the null value, indicating that the observed effect is not statistically significant. Individually, [14] reported an RR of 0.86 (95% CI: 0.68–1.09) with a weight contribution of 74.7%, while [12] showed a lower RR of 0.65 (95% CI: 0.42–1.01) and accounted for 21.8% of the overall analysis. The study by [13] had a smaller sample and higher uncertainty, with an RR of 1.16 (95% CI: 0.39–3.46), contributing only 3.6% to the total weight. Despite these variations, the heterogeneity remained low (I^2^ = 0.0%, τ^2^ = 0, *p* = 0.4489), indicating consistency across studies. While the results align with a trend favoring finerenone, the wide confidence intervals highlight the uncertainty surrounding its definitive effect on ESKD.

**Figure 4:**
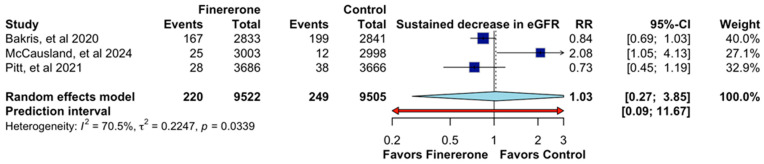
Effect of Finererone on Sustained decrease in eGFR. Data from Bakris et al., 2020 [14], McCausland et al., 2024 [13], and Pitt et al., 2021 [12].

A supplementary analysis was performed to examine the effect of finerenone on the continuous reduction in estimated glomerular filtration rate (eGFR), as shown in Figure 4. Data from all three studies were incorporated into this analysis [12,13,14], which included 19,027 participants. The pooled relative risk (RR) was 1.03 (95% CI: 0.27–3.85; Figure 4), indicating no significant difference between the finerenone and control groups with respect to the reduction in eGFR. This estimate also reveals a high degree of uncertainty in the prediction range (0.09–11.67). In the individual analysis, the study by [14] showed an RR of 0.84 (95% CI: 0.69–1.03), contributing the most to the overall analysis (40.0%). Ref. [12] reported a RR of 0.73 (95% CI: 0.45–1.19), contributing 32.9%. On the other hand, ref. [13] reported a RR of 2.08 (95% CI: 1.05–4.13), suggesting a potential increased risk, albeit with a wide margin and a 27.1% share. Importantly, the variability between investigations was significant (I2 = 70.5%, *p* = 0.0339), indicating variability in the findings.

This suggests that differences in study populations, definitions of sustained eGFR decline, or follow-up durations may have influenced the results. The high degree of heterogeneity reduces confidence in the pooled estimate, highlighting the need for further research to clarify the impact of finerenone on renal function decline in patients with chronic kidney disease and type 2 diabetes.

**Figure 5:**
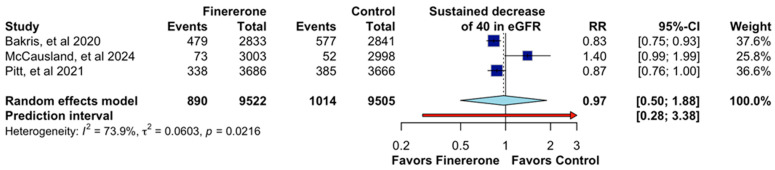
Effect of Finererone on Sustained decrease of 40 in eGFR. Data from Bakris et al., 2020 [14], McCausland et al., 2024 [13], and Pitt et al., 2021 [12].

The evaluation of the sustained decrease of 40 in eGFR, presented in Figure 5, included data from three randomized controlled trials [12,13,14], with a total of 19,027 patients. The pooled relative risk (RR) was 0.97 (95% CI: 0.50–1.88), indicating no statistically significant difference between finerenone and the control group regarding this renal outcome. The wide confidence interval suggests substantial uncertainty, and the prediction interval (0.28–3.38) further reflects the variability in the potential effect. Among individual studies, ref. [14] reported an RR of 0.83 (95% CI: 0.75–0.93) with the highest weight contribution (37.6%), followed by [12] with an RR of 0.87 (95% CI: 0.76–1.00) and a weight of 36.6%. In contrast, ref. [13] showed an RR of 1.40 (95% CI: 0.99–1.99) with a weight of 25.8%, suggesting a possible increased risk, though with a confidence interval crossing unity. Notably, heterogeneity was high (I^2^ = 73.9%, *p* = 0.0216), indicating considerable variability across studies. This level of heterogeneity suggests that differences in study design, patient populations, or treatment protocols may have influenced the results.

**Figure 6:**
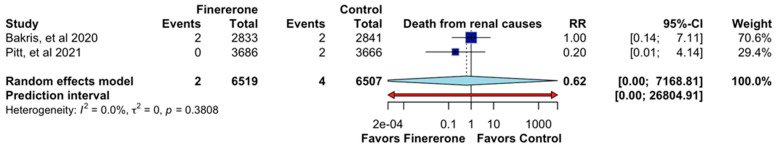
Effect of Finererone on Death from renal causes. Data from Bakris et al., 2020 [14], and Pitt et al., 2021 [12].

The analysis of death from renal causes, presented in Figure 6, included data from two randomized controlled trials [12,14], with 13,026 participants. The pooled relative risk (RR) was 0.62 (95% CI: 0.00–7168.81), indicating extreme uncertainty due to the very low number of events in both groups (two cases in the finerenone group and four in the control group). The broad confidence and prediction intervals (0.00–26804.91) reflect a lack of statistical power to determine a transparent effect. The absence of heterogeneity (I^2^ = 0.0%, *p* = 0.3808) suggests consistency across studies, but the overall rarity of renal mortality limits the interpretability of this outcome.

**Figure 7:**
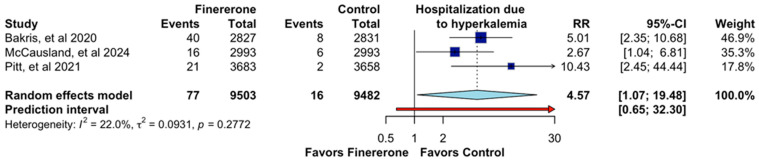
Effect of Finererone on Hospitalization due to hyperkalemia. Data from Bakris et al., 2020 [14], McCausland et al., 2024 [13], and Pitt et al., 2021 [12].

In Figure 7, the effect of finerenone on hospitalization due to hyperkalemia was evaluated, including data from [12,13,14], with a total of 18,985 participants. The pooled RR was 4.57 (95% CI: 1.07–19.48), indicating a significantly higher risk of hospitalization for hyperkalemia in patients receiving finerenone. The confidence interval suggests uncertainty regarding the exact magnitude of the effect, with a prediction interval of 0.65–32.30. Individual study results showed RRs of 5.01 (95% CI: 2.35–10.68) for [14], 10.43 (95% CI: 2.45–44.44) for [12], and 2.67 (95% CI: 1.04–6.81) for [13]. Despite some variability, heterogeneity remained low (I^2^ = 22.0%, *p* = 0.2772), indicating a relatively consistent association across studies.

**Figure 8:**
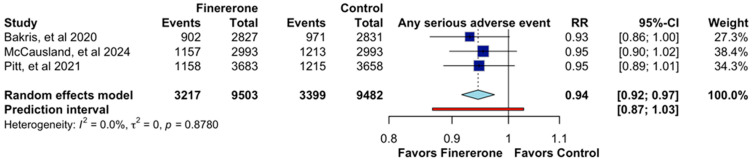
Effect of Finererone on any serious adverse event. Data from Bakris et al., 2020 [14], McCausland et al., 2024 [13], and Pitt et al., 2021 [12].

The analysis of serious adverse events, shown in Figure 8, included data from the same three trials. The pooled RR was 0.94 (95% CI: 0.92–0.97), suggesting a slight but non-significant reduction in serious adverse events with finerenone. The confidence interval does not indicate a meaningful protective effect, and the prediction interval (0.87–1.03) reinforces the lack of strong clinical significance. Heterogeneity was absent (I^2^ = 0.0%, *p* = 0.8780), confirming consistency in findings across studies.

**Figure 9:**
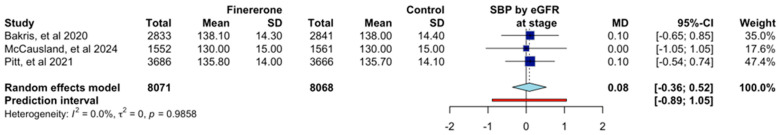
Effect of Finererone on SBP by eGFR at stage. Data from Bakris et al., 2020 [14], McCausland et al., 2024 [13], and Pitt et al., 2021 [12].

Finally, Figure 9 evaluated the effect of finerenone on systolic blood pressure (SBP) stratified by the eGFR stage. The pooled mean difference (MD) was 0.08 mmHg (95% CI: −0.36 to 0.52), indicating no significant effect of finerenone on SBP. The prediction interval (−0.89 to 1.05) further supports the absence of a clinically relevant difference between groups. Individual studies showed small and inconsistent changes in SBP, with mean differences of 0.10 mmHg (95% CI: −0.65 to 0.85) for [14], 0.10 mmHg (95% CI: −0.54 to 0.74) for [12], and 0.00 mmHg (95% CI: −1.05 to 1.05) for [13]. Heterogeneity was non-existent (I^2^ = 0.0%, *p* = 0.9858), reinforcing the consistency of findings.

## 4. Discussion

The findings of this meta-analysis contribute to the growing body of evidence supporting the role of finerenone in patients with chronic kidney disease (CKD) and type 2 diabetes (T2D). By consolidating data from multiple randomized controlled trials (RCTs), our study reinforces the renal protective effects of finerenone. However, it also highlights specific areas where results remain inconclusive or require further investigation.

### 4.1. Comparison with Previous Studies

Our results align with key clinical trials such as FIDELIO-DKD, which demonstrated that finerenone significantly reduced the risk of CKD progression and cardiovascular events in patients with albuminuric CKD and T2D [14]. In our meta-analysis, the pooled relative risk (RR) for kidney failure was 0.86 (95% CI: 0.35–2.13), suggesting a potential benefit, albeit with a wide confidence interval. Similarly, the FIGARO-DKD trial found that finerenone reduced cardiovascular risk, particularly in preventing hospitalization for heart failure. Our analysis also supported this finding [9]. One of the key differences observed in our study compared to FIDELIO-DKD was the variability in effect sizes for kidney failure and estimated glomerular filtration rate (eGFR) decline. In FIDELIO-DKD, finerenone demonstrated a hazard ratio (HR) of 0.82 (95% CI: 0.73–0.93, *p* = 0.001) for the primary composite kidney outcome, whereas our findings, although trending in the same direction, showed wider confidence intervals, reflecting potential heterogeneity among studies and patient populations. This discrepancy may stem from differences in baseline kidney function, albuminuria levels, and study follow-up durations.

Differences in baseline eGFR and albuminuria levels among the study populations may have contributed to the variation in the observed effects. Future clinical trials should present stratified analyses to determine whether the benefits of finerenone are greater in specific subgroups, such as patients with eGFR < 60 mL/min/1.73 m^2^ or UACR > 300 mg/g.

Additionally, our results suggest that finerenone does not significantly impact blood pressure reduction, as evidenced by our findings for systolic blood pressure (SBP) changes across different baseline eGFR categories. This is in agreement with previous research that shows that the renoprotective action of finerenone transcends its antihypertensive action [15] In contrast to the action of the steroidal mineralocorticoid receptor antagonists (MRAs) spironolactone, which is mainly by reduction of BP, finerenone’s action is thought to be a direct reduction of inflammation and fibrosis in cardiac and renal tissues [16].

Finerenone’s mechanism of action has been the focus of extensive research [15]. The mineralocorticoid receptor (MR) is an important contributor to chronic kidney disease (CKD) and cardiovascular disease (CVD), mainly through stimulating inflammation, oxidative stress, and tissue fibrosis [4]. Excessive MR activity may aggravate proteinuria, accelerate glomerulosclerosis, and lead to an increased risk of cardiovascular comorbidities, making MR an attractive target for a therapeutic approach [17].

Finerenone is a nonsteroidal mineralocorticoid receptor antagonist (MRA) with greater receptor selectivity and diminished risk of hyperkalemia compared with the steroidal MRAs (e.g., spironolactone and eplerenone). This is especially applicable to CKD patients, where increased potassium levels are a common dose-limiting factor to MRA use. Previously, the FIDELIO-DKD trial had shown that, although hyperkalemia occurred more frequently in finerenone-treated patients than placebo, the overall incidence of treatment discontinuation due to hyperkalemia was low, being 2.3% compared to 0.9% [14]. Our data support this risk but imply that regular monitoring and dose adjustment of potassium may alleviate this issue.

Interestingly, recent studies suggest that sodium-glucose cotransporter 2 inhibitors (SGLT2i) may reduce the risk of hyperkalemia when coadministered with mineralocorticoid receptor antagonists. This potential synergistic effect warrants further investigation in the near future, as it could improve the safety profile of finerenone in patients with chronic kidney disease and type 2 diabetes.

### 4.2. Clinical Implications and Patient Selection

Given its demonstrated benefits in reducing CKD progression and cardiovascular risk, finerenone represents an important therapeutic option for patients with moderate to severe CKD and T2D, particularly those already on optimal renin-angiotensin system (RAS) blockade. However, the optimal patient profile for finerenone therapy remains an active research area.

The FIDELITY analysis, which pooled data from FIDELIO-DKD and FIGARO-DKD, suggested that the benefits of finerenone extend across different levels of kidney function and are consistent regardless of baseline heart failure status [18]. Our study supports this notion but highlights substantial heterogeneity, particularly in eGFR decline, which may indicate that individual patient characteristics such as baseline albuminuria and blood pressure levels influence treatment response.

Interestingly, recent analyses have explored the synergistic effects of finerenone with sodium-glucose cotransporter-2 inhibitors (SGLT2is) [18]. Both drug classes exhibit complementary nephroprotective mechanisms, with SGLT2is reducing glomerular hyperfiltration and finerenone attenuating inflammatory and fibrotic pathways [19]. Preliminary evidence suggests that combining these therapies may further reduce the risk of CKD progression and cardiovascular events [19]. However, large-scale trials evaluating this combination are still needed. Recently, the CONFIDENCE study was published, and it ultimately significantly raises the question of this current strategy. CONFIDENCE is a phase 2 randomized, controlled trial evaluating the concomitant administration of finerenone and empagliflozin in patients with CKD and T2DM. The study suggested that this combination therapy achieved a 52% reduction in urinary albumin-creatinine ratio (UACR) at day 180, a value significantly greater than the reduction achieved with either agent alone. Specifically, the UACR reduction was 29% greater compared with finerenone monotherapy and 32% greater than empagliflozin alone [20] These results support the synergistic interaction between the two therapies and provide preliminary evidence that combined early intervention may improve renal protection in high-risk patients. Although long-term studies are needed, the CONFIDENCE trial provides promising insights into the future of combined cardiorenal therapy [20].

### 4.3. Limitations and Future Directions

Although this meta-analysis has the necessary methodological strengths, including a rigorous approach and the assessment of clinically relevant outcomes, we acknowledge the existence of certain limitations. Specifically, these are related to three aspects: the existence of heterogeneity among the included studies, the relatively short duration of follow-up, and the limited generalizability to broader populations, which may affect the interpretation and applicability of the results. Furthermore, future studies should explore dose-response relationships to improve the precision of clinical recommendations. The diversity of the studies included was one of the main obstacles faced. Variability in the estimated effects may have resulted from variations in patient groups, baseline characteristics, follow-up periods, and definitions of clinical outcomes. Even though statistical techniques were used to take this heterogeneity into consideration, residual confounding cannot be completely ruled out. The very short follow-up period of the included trials is another important restriction.

Over many years, chronic kidney disease (CKD) develops as a progressive illness. Even though the current investigation shows notable short- to medium-term effects, it is yet unknown if finerenone’s advantages last for longer. To ascertain if the noted decline in cardiovascular events and kidney failure translates into long-term, maintained protection, longer follow-up research is required.

Another potential mixing factor in lots of looked-at studies is side-by-side treatment. It was hard to separate the unique impacts of finerenone since a large part of the patients were using sodium-glucose cotransporter-2 inhibitors SGLT2is renin-angiotensin system RAS inhibitors, and other heart-protective drugs. Future studies should look into how finerenone works with these drugs to make the best treatment plans for people who have type 2 diabetes (T2D) and chronic kidney disease (CKD), though this is an echo of real-life prescribing habits. Several important topics for further research should take priority in light of these limitations. First, identifying the best patient profile for finerenone therapy is critical, particularly for those with high cardiovascular risk but with renal function. By determining which subgroups would benefit from finerenone the most, treatment recommendations can be improved, and patient outcomes can be enhanced.

Second, long-term trials are needed to see if the short-term benefits on the heart and kidneys translate into long-term reductions in kidney failure and death. For finerenone to go big in clinical use, knowing how long its effects last will be key. Possible synergy between finerenone and new treatments, namely SGLT2is and GLP-1 receptor agonists, constitutes another important line of inquiry. The combination treatment might bring additive or synergistic benefits to further reduce chronic kidney disease progression and its cardiovascular outcomes due to these drugs having different mechanisms of nephroprotection. Large-scale studies are needed to evaluate these combination approaches. Finally, studies need to be done to see if using finerenone makes sense money-wise. Its effectiveness is supported by clinical trial data, but wider adoption will depend on knowledge of its cost and accessibility in various healthcare settings.

## 5. Conclusions

This meta-analysis gives increasing evidence that finerenone should be an important treatment choice for people with CKD and T2D. Its capacity to lower the failure of the kidneys is reiterated by the results, and thus its potential to improve outcomes the traditional way beyond therapy approaches. Overall, the results indicate that finerenone is a beneficial advancement in chronic kidney disease treatment, even though some of the heterogeneity assessed outcomes was assessed. Finerenone will likely be a major element of modern CKD therapy as more studies shift its role toward cardiorenal protection. To improve its clinical impact, future studies should look at combination therapies, long-term outcomes, and optimizing patient selection and cost-effectiveness. From a clinical perspective, the ideal candidate for finerenone therapy would be a patient with type 2 diabetes, moderate to severe chronic kidney disease, persistent albuminuria, and a low baseline risk for developing hyperkalemia. Given the rising prevalence of CKD, finerenone is a potential medication that could change the way high-risk patients are treated.

## Figures and Tables

**Table 1:** Characteristics of included studies.

Author	Year	Country	Design	Number of Patients	Diagnostic Criteria	Duration of Follow-Up	Inclusion Criteria	Comorbidities
Bakris, et al. [14]	2020	Multinational study conducted in 48 countries.	RCT, parallel	Finererone = 2833 Placebo = 2841	CKD was defined as persistent moderate albuminuria (30 to <300) or severe albuminuria (300 to 5000) with an estimated glomerular filtration rate (eGFR) between 25 and 75 mL/min/1.73 m^2^.	Median follow-up: 2.6 years. Trial visits: Month 1, Month 4, then every 4 months until trial completion. Mean adherence to trial regimen: 92.1% finerenone group, 92.6% placebo group.	Adults (≥18 years) with type 2 diabetes and CKD. Persistent moderately elevated albuminuria (≥30 and <300 mg/g) with eGFR ≥25 and <60 mL/min/1.73 m^2^ and diabetic retinopathy. Persistent severely elevated albuminuria (300–5000 mg/g) with eGFR ≥25 and <75 mL/min/1.73 m^2^. Treated with ACE inhibitors or ARBs at maximum dose tolerable without unacceptable side effects.	Duration of diabetes: Mean 16.6 years (±8.8 SD). HbA1c: Mean 7.7% (±1.3 SD). eGFR: Mean 44.3 mL/min/1.73 m^2^ (±12.6 SD). Urinary albumin-to-creatinine ratio (UACR): Median 852 mg/g (IQR 446–1634).
McCausland, et al. [13]	2024	North America, Europe, Asia, and Latin America	RCT, parallel	Finerenone: 3003 Placebo: 2998	A sustained ≥50% decline in estimated glomerular filtration rate (eGFR) from baseline. A sustained eGFR decline to <15 mL/min/1.73 m^2^. The initiation of long-term dialysis or kidney transplantation.	Median of 2.6 years. The measurements were conducted at the following time points: baseline, 1 month, 3 months, 6 months, 12 months, 16 months, 20 months, 24 months, 28 months, 32 months, and 36 months.	Age: Patients aged 40 years or older. Heart failure diagnosis: Symptomatic heart failure with mildly reduced or preserved ejection fraction (LVEF ≥40%). Structural heart disease evidence: Left ventricular hypertrophy or left atrial enlargement within the previous 12 months. Elevated natriuretic peptides: Increased serum levels of natriuretic peptides (e.g., NT-proBNP) as an indicator of heart failure.	History of Hypertension: Total (Placebo): 87.3% Total (Finerenone): 85.3% History of Diabetes: Total (Placebo): 40.0% Total (Finerenone): 41.3% History of Myocardial Infarction: Total (Placebo): 25.3% Total (Finerenone): 26.5%
Pitt, et al. [12]	2021	Multinational study conducted in 48 countries.	RCT parallel, phase 3 clinical trial.	7437 (3686 finerenona, 3666 placebo)	CKD was defined as: 1. Moderately elevated albuminuria (urinary albumin-to-creatinine ratio ≥30 and <300 mg/g) and eGFR ≥25 and ≤90mL/min/1.73 m^2^ (stage 2–4 CKD). 2. Severely elevated albuminuria (urinary albumin-to-creatinine ratio 300 to 5000 mg/g) and eGFR ≥60 mL/min/1.73 m^2^ (stage 1–2 CKD).	Median follow-up was 3.4 years.	1. Adults aged ≥18 years with type 2 diabetes and CKD. 2. CKD defined as: - Persistent moderately elevated albuminuria (urinary albumin-to-creatinine ratio ≥30 and <300 mg/g) with eGFR ≥25 and ≤90 mL/min/1.73 m^2^ (stage 2–4 CKD). - Persistent severely elevated albuminuria (urinary albumin-to-creatinine ratio 300–5000 mg/g) with eGFR ≥60 mL/min/1.73 m^2^ (stage 1–2 CKD). 3. Treated with renin–angiotensin system (RAS) inhibitors at maximum tolerated dose.	45.3% with history of cardiovascular disease, 54.3% using insulin, 69.4% male. Other comorbidities: hypertension, obesity, and diabetic nephropathy.

**Table 2:** Characteristics of interventions of studies.

Characterictics of Intervention (Drug, Doses, Route, Frequency, etc.)	Content of Co-Intervention	Characterictics of Control (Drug, Doses, Route, Frequency, etc.)	Characteristics of Blinding	Other Treatments
Drug: Finerenone. Dose: 10 mg or 20 mg once daily. Route: Oral. Frequency: Once daily.	All patients were treated with renin–angiotensin system blockade (ACE inhibitors or ARBs) adjusted to the maximum tolerated dose before randomization.	- Drug: Placebo. - Dose: Not applicable. - Route: Oral. - Frequency: Once daily.	- Double-blind design: Neither participants nor investigators were aware of group assignments. - An independent committee adjudicated outcome events without knowledge of treatment assignments. - Medical writing assistance was blinded.	- Patients could receive glucose-lowering therapies (e.g., insulin, SGLT2 inhibitors), diuretics, statins, and potassium-lowering agents, as appropriate. - ACE inhibitors: 34.2%. - ARBs: 65.7%. - Statins: 74.3%. - Insulin: 64.1%.
Drug: Finerenone (non-steroidal mineralocorticoid receptor antagonist). Doses: 20 mg or 40 mg (adjusted according to baseline eGFR). Route: Oral. Frequency: Daily.	Standard therapies for heart failure and chronic kidney disease were allowed (e.g., ACE inhibitors, ARBs, diuretics, SGLT2 inhibitors).	Placebo.	Double-blind	
Drug: Finerenone, a selective nonsteroidal mineralocorticoid receptor antagonist. Doses: 10 mg or 20 mg once daily (based on eGFR). Route: Oral. Frequency: Once daily. Titration: Dose adjusted based on potassium levels and eGFR stability.	Renin–angiotensin system inhibitors (ACE inhibitors or ARBs) were required for all participants before and during the trial.	Drug: Placebo. Doses: Matched to finerenone dosing (10 mg or 20 mg once daily). Route: Oral. Frequency: Once daily.	Double-blind design. Patients, investigators, and sponsor were blinded to treatment assignments. Blinded clinical event committees adjudicated reported outcomes.	1. Sodium–glucose cotransporter 2 (SGLT2) inhibitors: Used by 8.4% at baseline; an additional 15.8% initiated therapy during the trial. 2. Glucagon-like peptide-1 (GLP-1) receptor agonists: Used by 7.5% at baseline; an additional 11.3% initiated therapy during the trial. 3. Statins: Used by 70.5%.

**Table 3:** Risk of bias assessment.

Domain	Mc Causland 2024	Bakris 2020	Pitt 2021
D1. Bias in the randomization process	Some concerns	Low risk	Low risk
D2. Bias due to deviations from interventions	Low risk	Low risk	Low risk
D3. Bias due to missing outcome data	Low risk	Low risk	Low risk
D4. Bias in measurement of the outcome	Low risk	Low risk	Low risk
D5. Bias in selection of the reported result	Low risk	Low risk	Low risk
Overall risk of bias	Some concerns	Low risk	Low risk

## Data Availability

Data are contained within the article.

## Supplementary Materials

The following supporting information can be downloaded at: https://www.mdpi.com/article/10.3390/jcm14186355/s1, Table S1: Search strategy used in PubMed, Scopus, Web of Science, and Embase. Checklist S1: PRISMA 2020 Checklist for reporting of this systematic review.
